# Effect of Nanofiller on the Mechanical Properties of Carbon Fiber/Epoxy Composites under Different Aging Conditions

**DOI:** 10.3390/ma14247810

**Published:** 2021-12-17

**Authors:** Tian Yang, Shijian Lu, Da Song, Xianyong Zhu, Israpil Almira, Jiaan Liu, Ying Zhu

**Affiliations:** 1 Key Laboratory of Automobile Materials (Ministry of Education), College of Materials Science and Engineering, Jilin University, Changchun 130022, China; yangt1009@163.com (T.Y.); lusj20@mails.jlu.edu.cn (S.L.); songda19@mails.jlu.edu.cn (D.S.); amlsl1619@mails.jlu.edu.cn (I.A.); zhuying1619@mails.jlu.edu.cn (Y.Z.); 2 School of Mechanical and Aerospace Engineering, Jilin University, Changchun 130022, China

**Keywords:** carbon fiber, composites, carbon nanotubes, nanoclay, aging, mechanical properties

## Abstract

In this study, carbon fiber-reinforced epoxy composites (CFRPs) containing multi-walled carbon nanotube (MWCNT) and halloysite nanoclay were fabricated. The effects of these nanofillers (MWCNT and nanoclay) on the tensile and flexural properties of the CFRPs under different aging conditions were studied. These aging conditions included water soaking, acid soaking, alkali soaking, and thermal shock cycling. The experimental results showed that, after accelerated aging, the mechanical performance of the CFRPs decreased. The performance degradation in the soaking environment depends on the immersion temperature and immersion medium. High-temperature accelerated the aging behavior of the CFRPs, resulting in low strength and modulus. The CFRPs were more vulnerable to acid soaking and alkali soaking than water soaking. The MWCNT and halloysite nanoclay are beneficial to improve the immersion aging resistance of the CFRPs, and the additions of nanofillers delayed the performance degradation under immersion aging conditions. However, nanofillers hardly improve the aging resistance of the CFRPs under thermal shock cycling condition. The fracture morphologies were observed by scanning electron microscopy (SEM) to reflect the failure modes of the CFRPs under various aging conditions. Differential scanning calorimeter (DSC) and fourier transform infrared (FTIR) spectroscopy tests were used to estimate the changes in the chemical structures and properties of epoxy resin and its composites under different conditions.

## 1. Introduction

Carbon fiber-reinforced epoxy resin composites (CFRPs) have many advantages such as their lightweight, good mechanical properties, and long service life; therefore, they are widely used in many fields, including sports equipment, automobiles, aerospace, and vessels [1,2]. In these application fields, CFRPs often undergo different degrees of corrosion, limiting the further development of the CFRPs [3,4].

CFRPs are commonly subjected to harsh aging conditions, e.g., hygrothermal, seawater, thermal shock cycling, which causes the polymer to react with corrosive medium, thereby decreasing the mechanical properties [5,6]. Owing to hydrolysis, plasticization, and matrix swelling, the mechanical properties of the CFRPs are degraded under aging conditions. One of the methods to improve the aging resistance of CFRPs is to add nanofiller [3,7,8,9,10,11]. These nanofillers include carbon nanotubes (CNTs), reduced graphene oxide (RGO), nanoclay, SiO_2_, etc [7,8,9,10,11]. It is reported that epoxy resins are brittle, and that the addition of functionalized CNT increases their mechanical properties because it can form a stronger interface between the functionalized CNT and the epoxy matrix favoring the stress transfer and the participation of effective strengthening effect [8]. CNTs have unique chemical properties, strengths, aspect ratios, and surface structures, and therefore, the addition of CNTs into an epoxy resin can increase the chemical complexity of the epoxy resin and significantly increase the mechanical properties of a CFRP [12,13,14]. Nanoclay was used as a nanofiller for epoxy resins to improve their mechanical strength and thermal properties [15]; however, nanoclay must be added in an appropriate amount; if too much is added, the agglomerating of the nanoclay will be too strong, which will degrade the performance of the CFRPs [15,16]. Besides, the chemical structure and physical characteristics of nanoclay also determine the tendency to form agglomerates [17].

Some researches reveal that nanofillers support the aging resistances of carbon fiber-reinforced epoxy resin composites (CFRPs) and glass fiber reinforced epoxy resin (GFRPs) [17,18,19,20]. Manjunath et al. added the nanoclay into a GFRP and found that the nanoclay improved the tensile and flexural properties of a GFRP by delaying their strength reduction [17]. Julio et al. studied the effect of nanofillers on the interlaminar fracture resistance of a CFRP under seawater aging conditions. The addition of MWCNT and RGO increased the fracture toughness of a CFRP [18].

Although some studies have investigated the aging behavior of the CFRPs with nanofillers in deionized water or salt solution [5,18], there is still a lack of enough information on multiple aging factors, e.g., acid solution, alkali solution, or under thermal shock cycling condition; therefore, in this work, the influences of nanofillers (MWCNT and nanoclay) on the mechanical properties of the CFRPs under water soaking, acid soaking, alkali soaking, and thermal shock cycling were investigated. The mechanical property degradations of the CFRPs under different aging conditions were systematically studied, which is a commonly used method to evaluate the aging behavior of the composites [21,22,23,24,25], and the related failure mechanisms were analyzed through fractographic observations by using SEM.

## 2. Materials and Methods

### 2.1. Preparation of the Composites

The raw materials used in this study were unidirectional carbon fiber fabric (CF, ZFS Materials Co., Ltd., Wuxi, China) and bisphenol-A E51 resin (EP, Nanton Synthetic Materials Co., Ltd., Wuxi, China).The weight of the carbon fiber is 300 g/m^2^, and the thickness is 0.167 mm.

Surface-treated MWCNTs were purchased (Zhongke Nano Materials Co., Ltd., Chengdu, China), and they were treated with a mixture of sulfuric acid and nitric acid (3:1), which introduced oxidation groups on the CNTs. The MWCNTs were stirred evenly in an acetone solvent for 2 h to obtain MWCNT acetone dispersion. Then, the MWCNT acetone dispersion was added to an E51 solution to achieve 0.5 wt.% content and sonicated at about 60 °C for another 2 h under a power of 40 kHz.

Surface-treated halloysite nanoclay (Mingchuang Materials Co., Ltd., Xi’an, China) was added to an appropriate amount of epoxy resin to achieve 2 wt.% content under stirring for 2 h at 25 °C. Then, the nanoclay-resin mixture was taken out.

**Figure 1:**
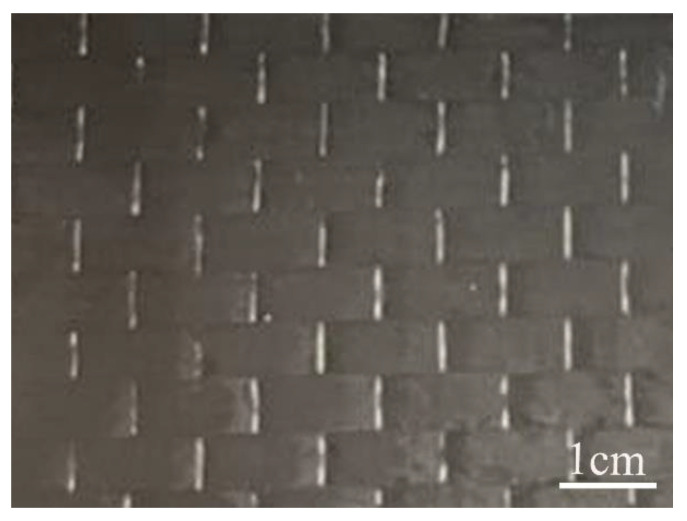
The details of the unidirectional CFRPs.

The resin/nanofiller mixture was infiltrated into CF fabric to prepare a prepreg. The prepreg was placed in the air for 72 h and then dried in a vacuum oven at 60 °C for 2.5 h. The unidirectional CFRP laminates were prepared by a molding process at a pressure of 1.1–1.3 MPa. The thickness of the CFRP laminates is about 2 mm. The details of unidirectional CFRPs are shown in Figure 1.

### 2.2. Aging and Thermal Shock Experiment

The soaking solutions used for accelerated aging tests were distilled water at 20 °C and 60 °C, HCl solution (5 wt.%) at 20 °C, and NaOH solution (10 wt.%) at 20 °C for an immersion time of 720 h. These soaking temperatures are usually applied for aging conditions [4,7]. The samples were taken out after soaking and dried in a vacuum oven at 40 °C for 48 h to remove water. Afterward, the samples were placed at room temperature for 120 h. Different aged samples were taken out and used for subsequent mechanical tests.

The different types of samples were used for thermal shock cycle tests. The temperature of the thermal shock test ranged from 0 °C to 100 °C. One thermal shock cycle included two steps, in which samples were first placed in a cold chamber at 0 °C for about 3 min and then set in a hot chamber at 100 °C for about 7 min. The experiment was repeated for 100 cycles, and then, the samples were dried for subsequent mechanical test.

### 2.3. Characterization and Mechanical Tests

The samples for tensile experiments were prepared and the experiments were conducted according to ASTM D3039. The samples with a size of 80 mm × 12.5 mm × 2 mm for three-point bending experiments were prepared and the experiments were conducted according to ASTM D7264. These two standard methods are often used for high-performance fibers/polymer composites [26,27,28,29,30]. More than five parallel specimens were tested in each group and an average value was calculated and used in this study.

After the mechanical tests, the failure morphologies of the samples were observed by scanning electron microscopy (SEM, Evo-18 Zeiss, Oberkochen, Germany) with an acceleration voltage of 15–20 kV. The sample was sprayed with gold before SEM observations using a deposition current of 15 mA for 4 min. The details of the MWCNT and nanoclay were examined by a transmission electron microscope (TEM, TECNAI F20, Hillsboro, OR, USA).

Fourier transform infrared spectroscopy (FTIR, Thermo Scientific., Waltham, MA, USA) was used for neat epoxy resin, epoxy resin-MWCNT composites, and epoxy resin-nanoclay composites to investigate the molecular changes in the epoxy system in the region of 500–3700 cm^−1^ with a resolution of 4 cm^−1^.

A differential scanning calorimeter (DSC) with Q2000 model was used for thermal analysis to determine the glass transition temperature (Tg) of the different materials. The heating rate was 10 °C/min.

## 3. Results and Discussion

### 3.1. Morphology of the Nanofillers

**Figure 2:**
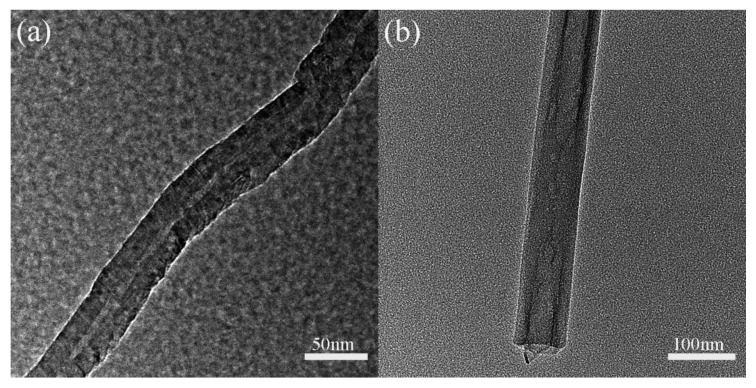
TEM images of (**a**) multi-walled carbon nanotube and (**b**) halloysite nanoclay.

The TEM images of MWCNT and halloysite nanoclay are shown in Figure 2. It can be seen that both MWCNT and nanoclay are tubular structures. The diameter of the MWCNT is ~40 nm and the length is 10–30 μm. The nanoclay has a diameter of ~60 nm and a length of 500–1000 nm. The high aspect ratio of these nanofillers is thought to improve the mechanical strength of the resin matrix by enhancing the nanofiller/resin interaction surface vicinity [12,13,14,15,16].

### 3.2. Tensile Properties

**Figure 3:**
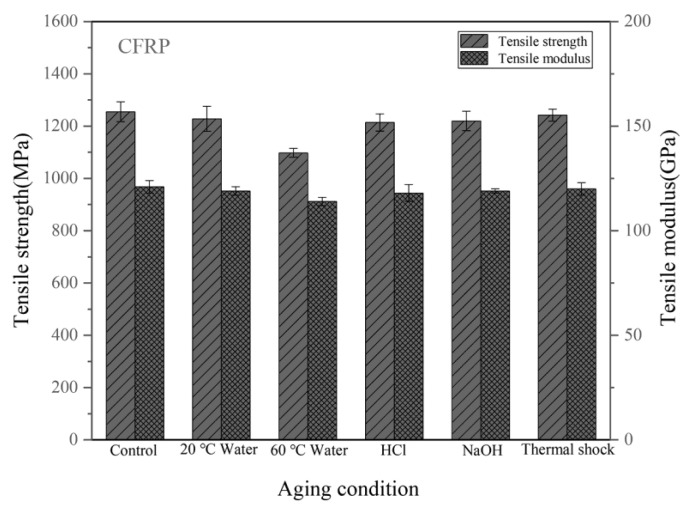
Tensile properties of the CFRPs without nanofiller under different aging conditions.

Figure 3 shows the tensile properties of the CFRPs without nanofiller under different conditions. For the neat CFRPs under a dry condition, the tensile strength was about 1255 MPa. It is evident from Figure 3 that the soaking aging behavior of the CFRPs is accelerated in a high-temperature environment, causing low strength and modulus of the CFRPs. In the hygrothermal environment, water molecules entered via the micropores of the resin matrix to form microcracks, which caused the matrix crack [18]. Hygrothermal aging at high-temperature reduced more mechanical properties because the epoxy resin absorbed more water in an elevated temperature environment. Therefore, the tensile strength of the CFRPs soaking in water at 60 °C is 1098 MPa, which is lower than those at 20 °C (1228 MPa). In addition, the soaking medium has a significant effect on the aging behavior of a CFRP. Acid soaking and alkali soaking at 20 °C led to the tensile strength of 1214 MPa and 1220 MPa, which are lower than water soaking at 20 °C. This indicates that the acid solution and the alkali solution have greater damage to the CFRP compared with water aqueous. It is reported that the acid entered the resin matrix via rapid penetration, where it swelled the matrix and the fiber-epoxy interface, causing plenty of cracks [31]. In the case of thermal shock cycling, the tensile properties of the aged sample are slightly lower than those of the control sample (1.04% in strength). The possible reason for that is a high-temperature in the hot chamber caused post-curing of the resin matrix, which increases the crosslinking and improves the mechanical properties, even though the thermal shock cycling also leads to a debonding effect at the interface [6].

**Figure 4:**
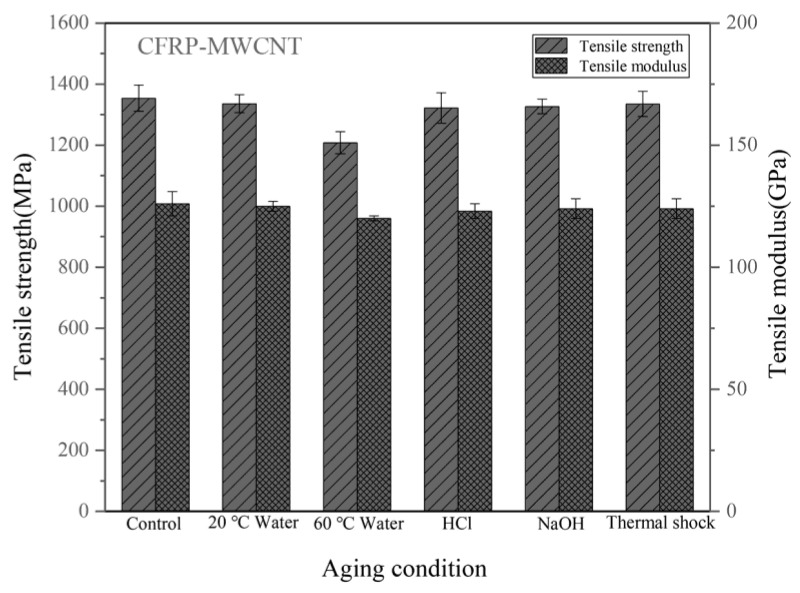
Tensile properties of the CFRPs with MWCNT under different aging conditions.

Figure 4 shows the tensile properties of the CFRPs with MWCNT under different conditions. Table 1 lists the tensile property reduction (%) of the CFRPs under different aging conditions. It can be seen that the unaged CFRPs containing 0.5 wt.% MWCNTs have the tensile strength of 1354 MPa. This indicates that the addition of MWCNT improved the mechanical strength of composites. The CNT increased its deformation resistance by increasing the toughness of the epoxy resin [32,33]. Besides, the functionalized CNT had a high interfacial adhesion, which also reduced the crack growth when bearing a load, causing an increase in strength [8,34]. After the aging test, the strength of the CFRPs with MWCNT also decreased and is depending on the soaking temperature and aging medium. A higher tensile strength of 1336 MPa is obtained in water soaking at 20 °C compared with a tensile strength of 1208 MPa in water soaking at 60 °C. Although the mechanical properties are reduced after hygrothermal aging, the presences of MWCNT help to reduce this negative effect. After water soaking at 60 °C, the MWCNT improved tensile strength and modulus by delaying the reduction rate under soaking conditions, indicating that MWCNT improved the soaking aging resistance. The CNT brings composites a low rate in performance reduction when the composites were exposed to an aging environment because the CNT is hydrophobicity and high barrier properties, and therefore, it hinders the penetration of water molecules [33,35,36]. In addition, in the case of thermal shock cycling, the MWCNT hardly improves the tensile properties; and the CFRPs with MWCNT show a higher reduction rate (1.40%) than the neat CFRPs (1.04%) in strength. The interactions of thermal shock on the mechanical strengths of the CFRPs are competitive [6]. On the one hand, a high-temperature caused the post-curing of the resin matrix, which increased the crosslinking and improved the mechanical properties. On the other hand, the thermal expansion coefficients of the composite components are different, leading to an interface debonding effect, which degraded the mechanical properties. In this study, the comprehensive effect from these competitive interactions could cause a reduction in strength during thermal shock cycling, which will be discussed by observing the fracture morphology of the CFRPs in the next section.

**Figure 5:**
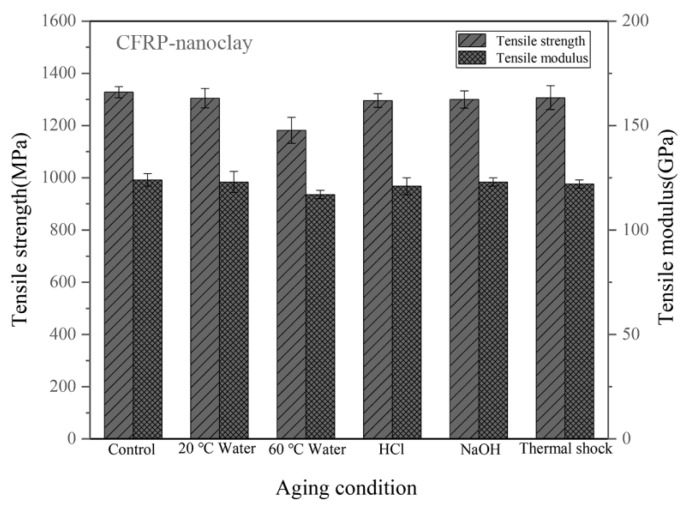
Tensile properties of the CFRPs with nanoclay under different aging conditions.

Figure 5 shows the tensile properties of the CFRPs with nanoclay under different conditions. The CFRPs with 2 wt.% nanoclay have tensile strength of 1328 MPa. In hygrothermal aging, the strength and modulus of the CFRP with nanoclay decline more in the high-temperature immersion. The tensile strength of the CFRP after soaking in water at 60 °C is 1182 MPa, which is lower than those after soaking in water at 20 °C (1305 MPa). Moreover, the nanoclay brings a visible improvement in the aging resistance to CFRP under the water soaking, acid soaking and alkali soaking at 20 °C; and the CFRPs with nanoclay are higher tensile properties and lower reduction rate in tensile properties than the neat CFRP. The reason for this is that the nanoclay could act as the barrier with the passageway of water molecules into the epoxy resin [18,37]. However, the nanoclay is not significantly improve the tensile strength under thermal shock cycling condition, and it can be seen from Table 1 that the CFRP with nanoclay has a higher reduction rate (1.58%) than the neat CFRP (1.04%).

### 3.3. Tensile Fracture of the CFRPs

**Figure 6:**
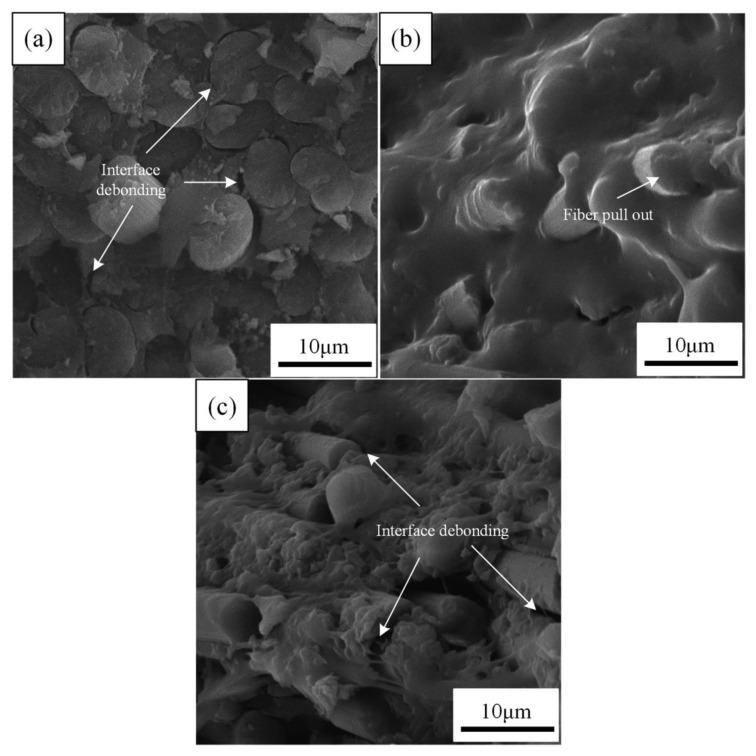
SEM images of the fracture surface of the unaged samples: (**a**) neat CFRPs, (**b**) CFRPs with MWCNT and (**c**) CFRPs with nanoclay.

Figure 6 shows the SEM images of the fracture surface of the unaged CFRPs. Figure 6a exhibits that the fracture surface of the unaged neat CFRP is flat and the CF/EP interface shows a visible debonding. Figure 6b displays the fracture surfaces of the samples with MWCNT are rough, and the epoxy resin is well bonded with the MWCNT. These results indicate that the addition of MWCNT is in favor of interface cohesion. It will reduce the stress concentration and allow CFRP to undergo high strain deformation, and they caused an increase in strength [36]. Figure 6c exhibits a moderate interface adhesion when nanoclay is applied in the CFRPs, because some resins adhere to the fiber surface. It has been reported that the nanoclay would keep the intensive transfer of load to fiber by increasing interfacial bonding and preventing crack propagation, leading to high mechanical properties [38].

**Figure 7:**
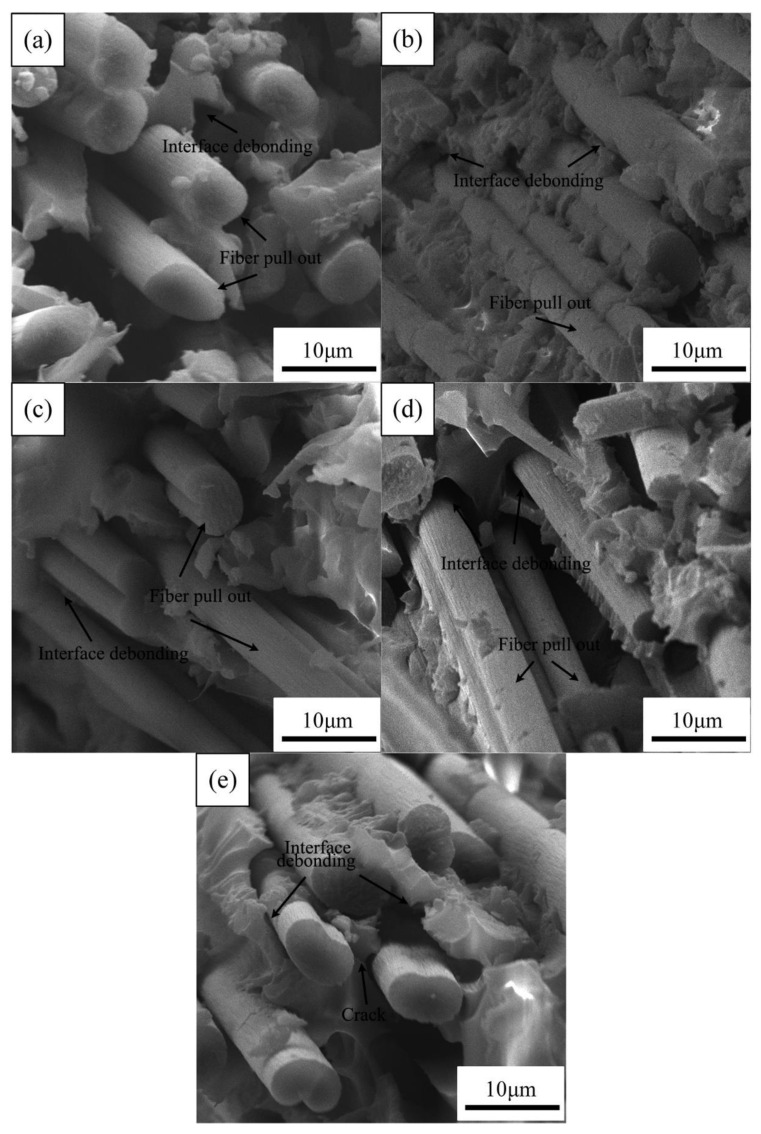
SEM images of the fracture surface of the aged neat CFRPs: (**a**) water soaking at 20 °C, (**b**) water soaking at 60 °C, (**c**) acid soaking, (**d**) alkali soaking, and (**e**) thermal shock cycling.

Figure 7 shows the SEM images of the fracture surface of the aged CFRPs without nanofillers. It is evident from Figure 7a,b that water entered via the micropores on the resin surface in different aqueous environments since water enters the resin to plasticize and swell the resin. Many fibers and epoxy resin were still tightly bound under water soaking conditions, which indicates moderate corrosion of the CFRPs when soaked in aqueous environments. In Figure 7c, when the CFRP was immersed in an acid solution, a large amount of resin disappeared. The hydrolysis of the resin was the main reason for the decrease in the mechanical properties of CFRP [34]. In Figure 7d, when soaked in an alkaline environment, the matrix underwent a large degree of hydrolysis, plasticization and swelling, which caused damage to the resin. Meanwhile, the interface was gradually eroded; thereby, fiber and resin were no longer tightly adhered [34,39]. It is evident from Figure 7e that the resin/fiber interface was debonded, and a small crack is growing from the interface to the resin matrix, which is one of the dominant failure modes during the thermal shock cycling [6].

**Figure 8:**
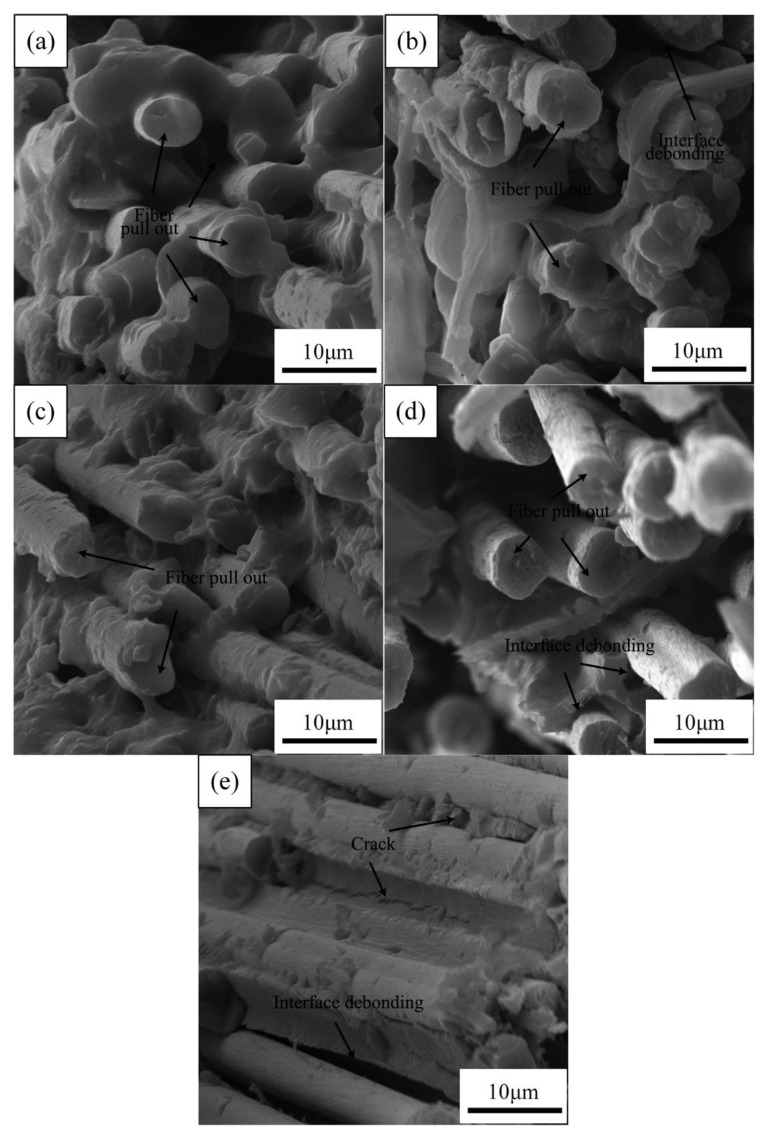
SEM images of the fracture surface of the aged CFRPs with MWCNT: (**a**) water soaking at 20 °C, (**b**) water soaking at 60 °C, (**c**) acid soaking, (**d**) alkali soaking, and (**e**) thermal shock cycling.

Figure 8 shows the SEM images of the fracture surface of the aged CFRPs with MWCNT. After adding MWCNT, the fracture surface of the CFRPs became rougher, and the fracture path increased. In addition, it can be seen from Figure 8a–c that the resin was a firmly adhesive carbon fiber surface, which indicates that the interface bond intensity increased, and such improved bond intensity promotes an incensement in mechanical strength of a CFRP in the same hygrothermal aging condition [17,40].

However, after the thermal shock cycling, interfacial debonding and resin microcracks were visible, as shown in Figure 8e. This might be attributed to plenty of interfaces introduced by CNT addition. These interfaces will be debonding under the action of thermal stress due to different thermal expansion coefficients of the composite components, which would be detrimental to mechanical properties.

**Figure 9:**
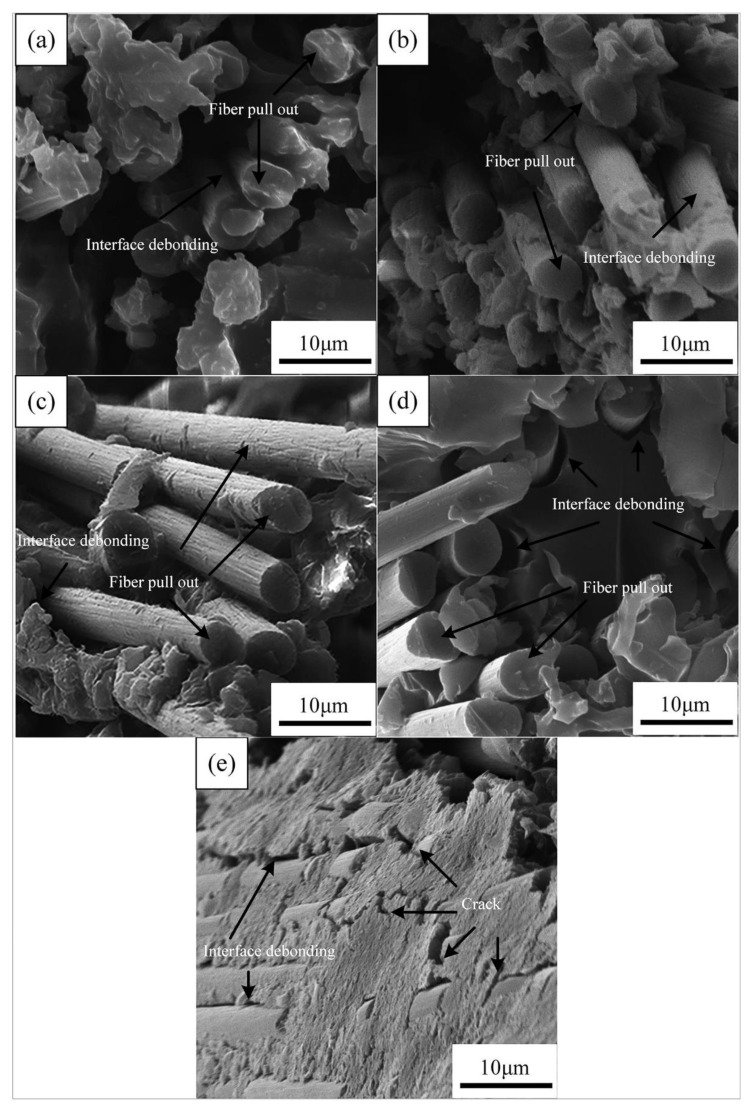
SEM images of the fracture surface of the aged CFRPs with nanoclay: (**a**) water soaking at 20 °C, (**b**) water soaking at 60 °C, (**c**) acid soaking, (**d**) alkali soaking, and (**e**) thermal shock cycling.

Figure 9 shows the SEM images of the fracture surface of the aged CFRPs with nanoclay. It can be seen from Figure 9a–d that the fracture surface of the sample with nanoclay was different from that of a neat sample. The CFRPs, by adding nanoclay, exhibited moderate interface adhesion, and, therefore, nanoclay addition reduced the stress concentration and improved the mechanical strength on the immersion conditions [18,37]. However, the nanoclay did not display a significantly improved effect during the thermal shock cycle. Figure 9e exhibits plenty of tiny cracks initiated at the interface. Resin crack and interface debonding are obvious; these failure modes observed by SEM lead to low mechanical properties and are responsible for the changes in mechanical properties of the CFRPs.

### 3.4. Flexural Properties

**Figure 10:**
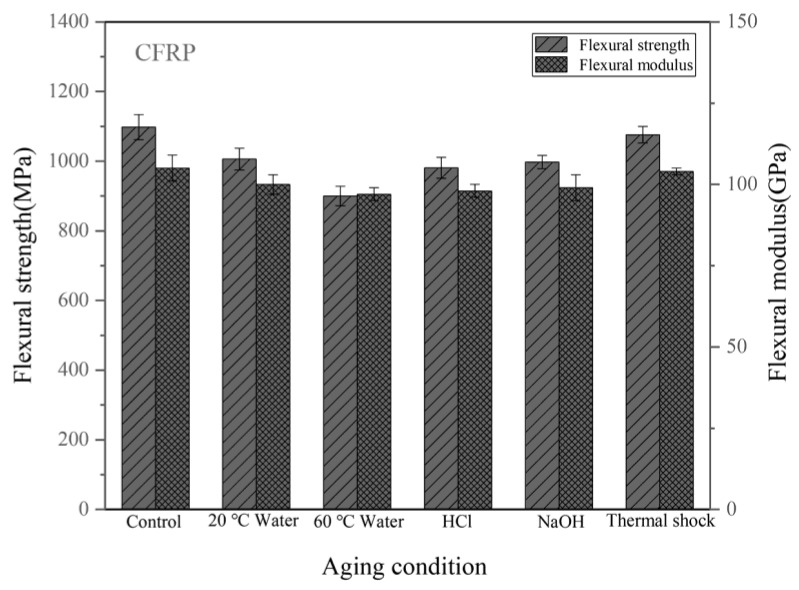
Flexural properties of the CFRPs without nanofillers under different aging conditions.

**Figure 11:**
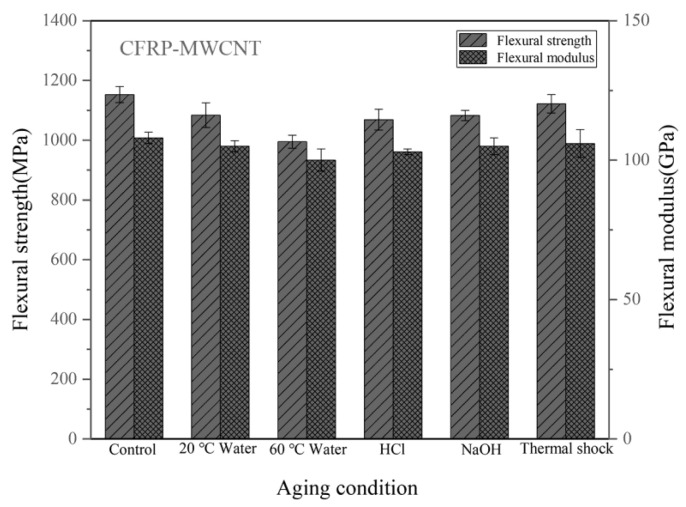
Flexural properties of the CFRPs with MWCNT under different aging conditions.

**Figure 12:**
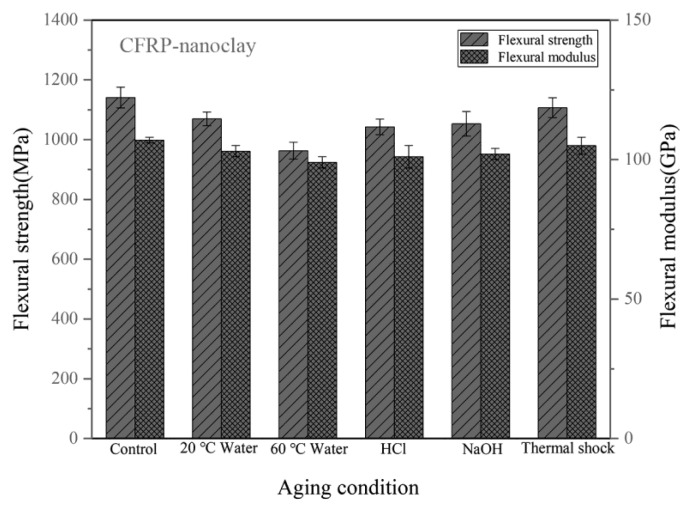
Flexural properties of the CFRPs with nanoclay under different aging conditions.

Figure 10 shows the flexural properties of the CFRPs without nanofillers. The strength of the unaged sample is 1098 MPa. Table 2 lists the flexural property reduction of the CFRPs under different aging conditions. After water immersion at 20 °C and 60 °C, the flexural strength decreased by 8.38%, and 18.03%, respectively. When soaking in acid and alkali solutions at 20 °C, the flexural strengths were both decreased. At these immersion conditions, the flexural modulus was reduced by 4.76%, 7.62%, 6.67%, and 5.71%, respectively. Figure 11 shows the flexural properties of the CFRPs containing MWCNT. After water immersion at 20 °C and 60 °C, the flexural strength decreased by 5.98% and 13.70%, respectively. When soaking in acid and alkali solutions at 20 °C, the flexural strengths were decreased to 1069 MPa and 1083 MPa, respectively. Figure 12 shows the flexural properties of the CFRPs containing nanoclay. Both strength and modulus were reduced after soaking aging. When soaking in water at 20 °C and 60 °C, the flexural strength decreased by 6.22%, and 15.60%, respectively. After acid and alkali immersion at 20 °C, the flexural strengths were reduced. Decreased mechanical properties are mainly attributed to hydrolysis, plasticization and swelling effects on resin matrix, which reduce the flexural properties of the CFRPs, and would cause further diffusion of water in the CFRPs. By comparing with the neat CFRPs, it is concluded that the CFRPs containing nanofillers exhibited greater hygrothermal aging resistance. Improved aging resistance might be due to the MWCNT and nanoclay that increased the effective surface area for a good interface adhesion and acted as a barrier to water molecules [17,40,41].

In the case of thermal shock cycling, the flexural strength decreased by 2.00%, 2.69% and 2.98% for neat CFRPs, MWCNT reinforced CFRPs and nanoclay reinforced CFRPs. Correspondingly, the flexural modulus decreased by 0.95%, 1.85% and 1.87% for neat CFRPs, MWCNT reinforced CFRPs and nanoclay reinforced CFRPs. These results also indicate that the nanofillers hardly improve the mechanical properties of the CFRP under thermal shock cycling condition.

### 3.5. Flexural Fracture of the CFRPs

**Figure 13:**
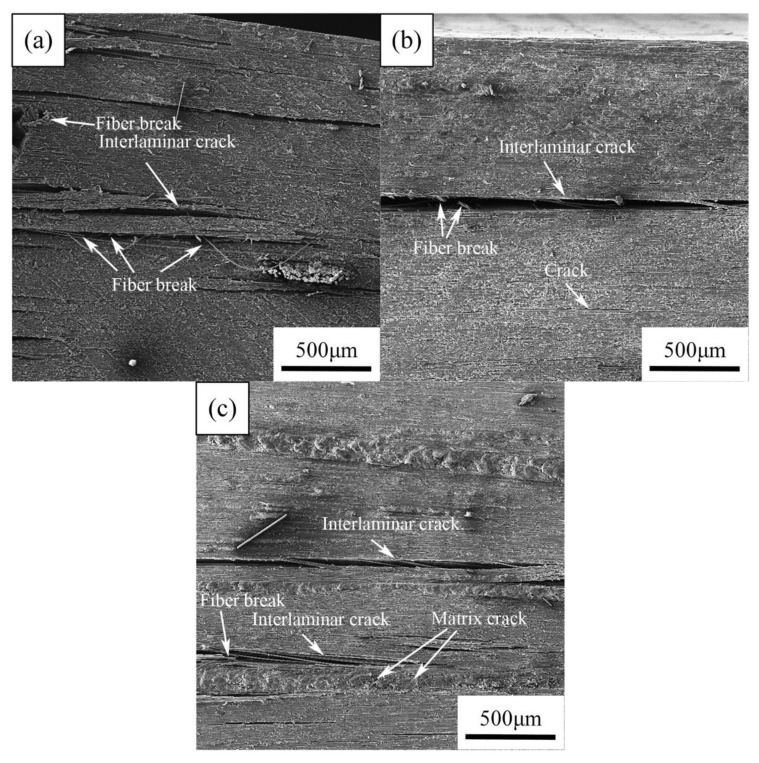
SEM images of the cross-section of the unaged CFRPs after flexural tests: (**a**) neat CFRPs, (**b**) CFRPs with MWCNT, (**c**) CFRPs with nanoclay.

Figure 13 shows SEM images of the fracture cross-section of the unaged CFRPs after flexural tests. All experimental CFRPs samples appeared large interlaminar cracks along with the longitudinal fiber directions. In addition, some fibers were breaking in the interlaminar of carbon fabric. These observations reflect a dominant failure mode of the experimental CFRPs during flexural tests is an interlaminar fracture, which is similar to previous studies [40,41]. In addition, Figure 13b reveals the MWCNT reinforced CFRPs have relatively fewer cracks compared with neat CFRPs. Moreover, the CFRPs with nanoclay are less damaged than neat CFRPs, as shown in Figure 13c. These might be the result of good interfacial adhesion between resin and fibers due to the presence of nanofillers.

### 3.6. DSC Analysis

**Figure 14:**
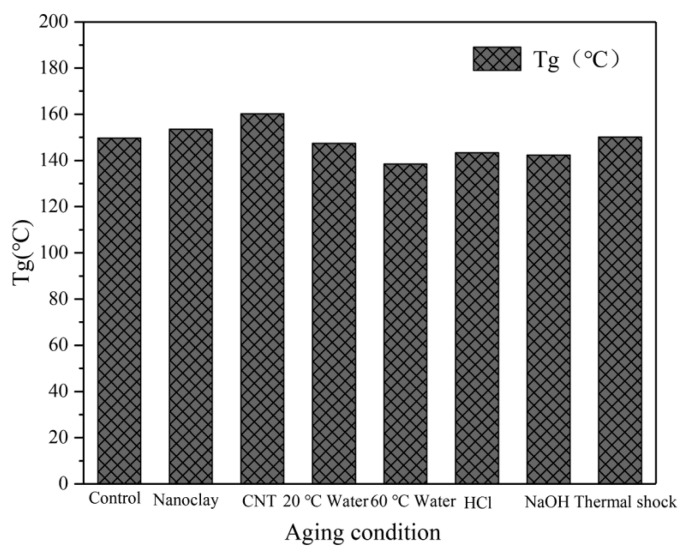
Tg of specimens under different conditions.

DSC tests for the neat and nanophased resin composites were used to determine the Tg of the experimental resin composites before and after aging. The Tg values of the resin composites under different conditions are shown in Figure 14. It can be seen that the values of Tg of resin composites containing MWCNT and nanoclay were higher than those of the neat resin. In addition, the soaking aging at high-temperatures brings Tg a slight decrease. After soaking in water at 20 °C and 60 °C, the Tg was 147.4 °C and 138.5 °C, respectively. When soaked in acid and alkali solutions at 20 °C, the Tg decreased to 143.4 °C and 142.3 °C, respectively, as compared with the initial Tg. Under hygrothermal conditions, water molecules will diffuse into the resin composites. The resin is easily corrosive and will absorb moisture. The penetration of water into the resin matrix increased the distance between the polymer segments and reduced the force between these segments, making the movement of segments easier, thus reducing the Tg [42,43]. The Tg value after thermal shock cycling was 150.1 °C. A possible reason for that is high temperature in the hot chamber during thermal cycling caused the post-curing of the resin matrix [44].

### 3.7. FTIR Analysis

**Figure 15:**
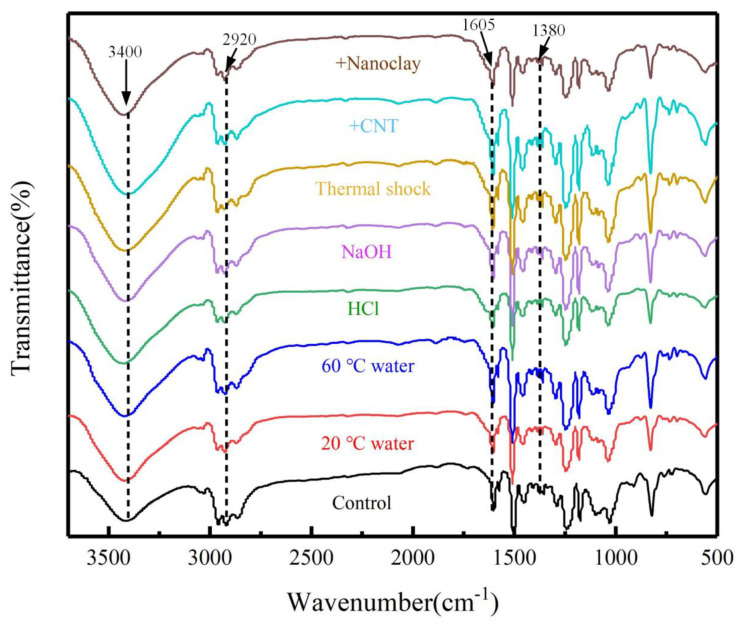
FTIR spectrum of specimens before and after aging.

Chemical structure analysis was conducted with FTIR. Figure 15 shows FTIR spectras of the neat epoxy resin, epoxy resin-MWCNT composites and epoxy resin-nanoclay composites to identify the structure change of the composites. The peaks at 3400 cm^−1^ for unaged and aged conditions corresponded to –OH group according to the previous study [45]. The peaks at 2920 cm^−1^ and 1605 cm^−1^ belonged to C–H bonds and C=C units, respectively [46,47]. The peaks at 1380 cm^−1^ were related to the –OH bending vibration from carboxyl –COOH groups [48]. Those peaks mentioned above are usually to estimate the influences of nanofiller and various aging conditions on the changes in the chemical structure of the resin composites [45,46,47,48,49,50]. It can be seen that those specific peaks of the epoxy resin composites containing MWCNT and nanoclay were almost identical to those of the neat epoxy resin, indicating that there was no new chemical matter that appeared when the nanofillers were added into the epoxy resin. Similar investigations were reported by previous studies [49,50]. To investigate the functional group changes of neat epoxy resin before and after aging, the peak locations and intensities peak at specific wavenumbers were further analyzed. It was noticed that the locations of the peaks of the 3400 cm^−1^, 2920 cm^−1^, 1605 cm^−1^ and 1380 cm^−1^ were not obviously changed, although the intensities of those peaks varied with the aging conditions.

## 4. Conclusions

In this work, the tensile and flexural properties of CFRP composites with nanofillers (MWCNT and nanoclay) were studied under different aging conditions. The conclusions are as follows:

(1) During immersion aging, the tensile and flexural properties of the CFRPs decreased. The aging behavior depends on the aging temperature and aging medium. High-temperature accelerated the mechanical property degradation of the CFRPs, resulting in low strength and modulus. The acid solution and the alkali solution more strongly damaged CFRPs compared with water aqueous at the same temperature.
(2) The addition of MWCNT improved the aging resistance of the CFRPs under soaking conditions due to a good interfacial interaction and high barrier property of MWCNT. The nanoclay brings an improvement effect on the aging resistance to CFRPs under soaking aging conditions, attributed to a high aspect ratio and moderate interface adhesion. As a result, the CFRPs containing nanofillers reduce less in tensile and flexural properties than neat CFRPs.
(3) The MWCNT and nanoclay hardly improve the aging resistance during thermal shock cycling. The SEM observation confirms several tiny cracks in the interface and resin under the action of stress due to different thermal expansion coefficients of the composite components during thermal shock cycling, which is one of the dominant failure mechanisms of the CFRPs containing nanofillers.

## Figures and Tables

**Table 1:** Tensile property reduction (%) of the CFRPs under different aging conditions.

Sample	Mechanical Properties	Water Soaking at 20 °C	Water Soaking at 60 °C	HCl Soaking	NaOH Soaking	Thermal Shock Cycling
CFRPs (without nanofiller)	Tensile strength	2.15	12.51	3.27	2.79	1.04
	Tensile modulus	1.65	5.79	2.48	1.65	0.83
CFRPs with MWCNT	Tensile strength	1.33	10.78	2.36	1.99	1.40
	Tensile modulus	0.79	4.76	2.38	1.59	1.59
CFRPs with nanoclay	Tensile strength	1.73	10.99	2.41	2.11	1.58
	Tensile modulus	0.81	5.65	2.42	0.81	1.61

**Table 2:** Flexural property reduction (%) of the CFRPs under different aging conditions.

Sample	Mechanical Properties	Water Soaking at 20 °C	Water Soaking at 60 °C	HCl Soaking	NaOH Soaking	Thermal Shock Cycling
CFRPs (without nanofiller)	Flexural strength	8.38	18.03	10.66	9.11	2.00
	Flexural modulus	4.76	7.62	6.67	5.71	0.95
CFRPs with MWCNT	Flexural strength	5.98	13.70	7.29	6.07	2.69
	Flexural modulus	2.78	7.41	4.63	2.78	1.85
CFRPs with nanoclay	Flexural strength	6.22	15.60	8.59	7.71	2.98
	Flexural modulus	3.74	7.48	5.61	4.67	1.87

## Data Availability

Not applicable.
